# Laparoscopic Management for Carcinoid Metastasis to the Spleen

**DOI:** 10.1155/2011/346507

**Published:** 2011-04-06

**Authors:** Damian Balmforth, Christos Skouras, Fausto Palazzo, Emmanouil Zacharakis

**Affiliations:** ^1^Department of Surgery and Cancer, Imperial College London, St. Mary's Hospital, London W21NY, UK; ^2^Department of Endocrine Surgery, Hammersmith Hospital, London W120NN, UK

## Abstract

We report a rare case of a laparoscopic splenectomy performed for a carcinoid metastasis. The patient represented with pleuritic left-sided chest pain from pleural deposits 9 years following resection of a primary lung carcinoid tumour. They were found to have a 4.7 cm splenic lesion on CT with a probable left acetabular metastasis demonstrated on Gallium PET scan. The patient underwent laparoscopic splenectomy for debulking treatment of the splenic lesion that was confirmed to be a splenic metastasis of the resected carcinoid lung tumour. Following an uncomplicated recovery, the patient was discharged on the second postoperative day. On discharge, she received adjuvant therapy with Lutetium 177 DOTATATE. This is the first report of a carcinoid splenic metastasis successfully treated with laparoscopic splenectomy.

## 1. Introduction

The spleen is a rare location of metastasis from solid tumours. In a recent review of 6,137 patients with metastatic malignant tumours, only 59 (0.96%) involved the spleen. In an earlier series of lung cancer patients, just 12 of 997 consecutive cases had splenic involvement. However, all 12 had other abdominal organ involvement in addition to the spleen at the time of presentation [1]. Carcinoid metastasis involving the spleen is extremely rare with only a few isolated cases being reported to date. Falk and Stutte and Eriguchi et al. report intraperitoneal spread of carcinoid tumours from ileal and gastric primaries, respectively, whilst Takada and Takami report on a case of isolated splenic metastasis of a primary lung carcinoid treated with traditional open splenectomy [2–4]. Due to the rarity of splenic metastasis, optimal management must be determined on an individual basis. In this case, the splenic metastasis of a primary lung carcinoid was successfully treated with laparoscopic splenectomy. Laparoscopic splenectomy is now considered to be the gold standard for treatment of benign haematological disease, with reduced morbidity and mortality compared to open procedures. However, evidence for its use in malignant disease of the spleen remains limited [5]. 

This case serves as a report of a rare event of metastatic bronchial carcinoid involving the spleen and its successful management with laparoscopic splenectomy.

## 2. Case Report

**Figure 1:**
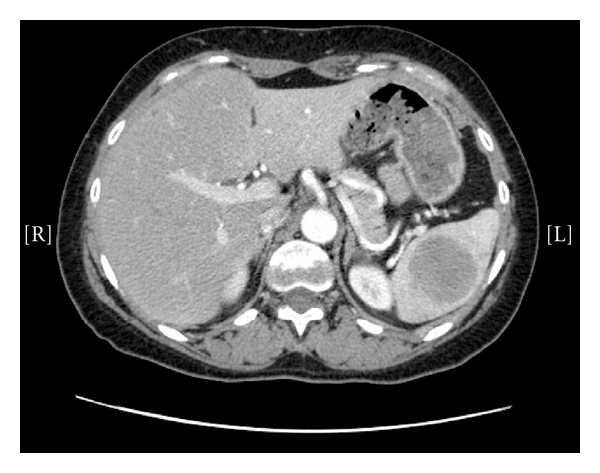
CT scan showing a 4.7 cm solitary splenic lesion.

A 51-year-old lady was admitted to our hospital in February 2009 with left-sided pleuritic chest pain. She had a history of left upper lobectomy in December 2000 for a primary carcinoid tumour of the lung (chromogranin positive; no mitotic activity; Ki67 labelling index of 2%; clear margins). The patient had been discharged from followup in 2005 with no evidence of recurrence. On representing in 2009, a CT scan showed evidence of local recurrence with left-sided pleural lesions and an enlarged left hilar lymph node, as well a solitary 4.7 cm splenic lesion (Figure 1). The patient was further investigated with Octreotide SPECT and Gallium 68 whole body PET studies, which showed uptake in the pleural lesions, no uptake of the splenic lesion, and normal appearance of the abdominal and pelvic viscera. However, increased activity was demonstrated in the left acetabulum, which was thought to represent bony metastasis. An ultrasound guided percutaneous biopsy of the splenic lesion diagnosed a low-grade neuroendocrine carcinoma in keeping with metastasis of the resected lung primary (chromogranin, cam 52, panCK, MFF116, synaptophysin, CD56 positive: no necrosis; no mitoses; Ki67 labelling index < 5%). After a multidisciplinary discussion, a funding application for Lutetium 177 DOTATATE therapy was made. Prior to the commencement of this therapy, a repeat CT scan in September 2009 showed a decrease in the size of the patient's pleural deposits, but an increase in the splenic lesion to 5.8 cm. In view of the isolated progression of the splenic lesion, it was decided that Lutetium therapy would be more effective following a reduction in the tumour load by excision of the splenic mass. As a result, the patient underwent a laparoscopic splenectomy in November 2009. 

**Figure 2:**
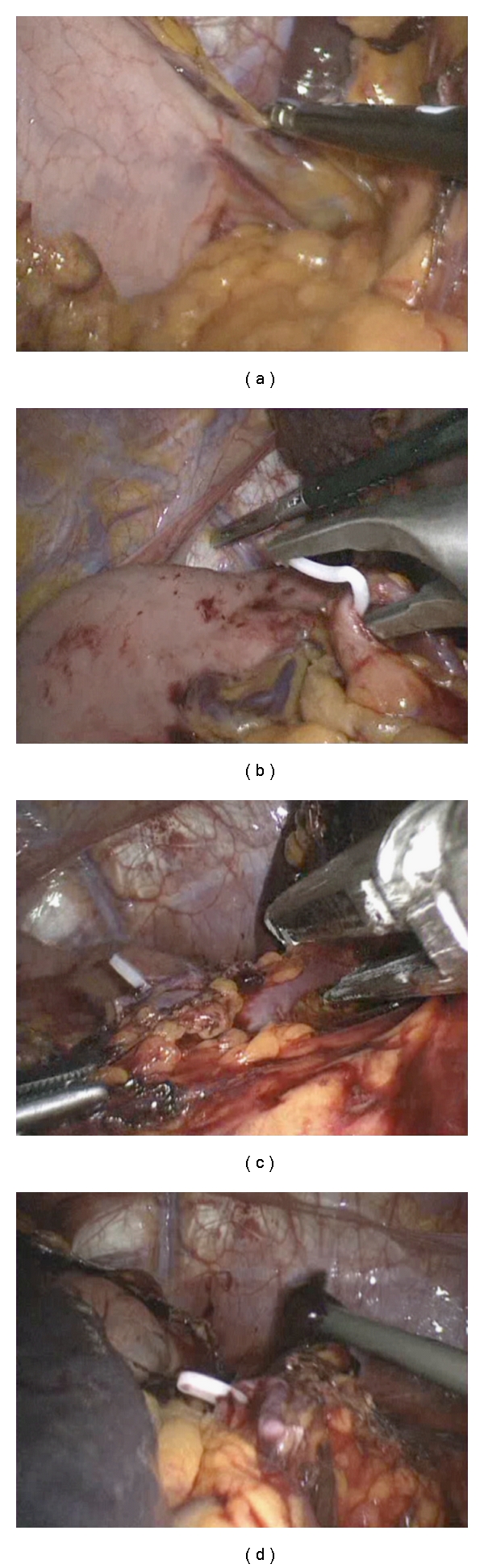
Intraoperative images of the performed laparoscopic splenectomy. (a) Ligation of the short gastric arteries. (b) Dissection and ligation of the splenic artery. (c) Ligation of the splenic hilum using the Ethicon stapler. (d) View of the operative field after completion of splenectomy.

The patient was placed in the right lateral decubitus position, and 4 ports were placed in the left flank. The colonic attachments were divided, and the short gastric vessels were controlled with the Ligasure vessel sealing system (Covidien, Valleylab, Boulder, CO, US) to allow isolation of the splenic hilum (Figure 2(a)). The splenic artery was then dissected and ligated with a hemostatic clip (Figure 2(b)). Finally, the splenic hilar vessels were dissected and divided using a vascular linear stapler (Ethicon Endo-Surgery, Inc. Cininnati, OH, US) (Figure 2(c)). Following complete splenic mobilisation (Figure 2(d)), the spleen was placed in a leak-proof bag and exteriorised to the abdominal wall. Histological analysis of the specimen confirmed a metastatic carcinoid tumour (chromogranin, cam 5.2, MFF116, synaptophysin, NSE and CD56 positive; no necrosis; Mitotic count 2 per HPF; Ki67 labelling index <5%). The patient made a good postoperative recovery and was discharged on the second postoperative day.

Since discharge the patient has been commenced on Lutetium 177 DOTATATE therapy. Prior to commencement, repeat PET scan in April 2010 showed the formation of several liver metastases. The patient's pleural disease had also increased whilst her bony acetabular lesion was unchanged in appearance. The patient has since completed 3 cycles of Lutetium therapy and her latest Gallium PET scan from October 2010 shows little disease progression. Clinically, the patient has no symptoms of carcinoid syndrome and but suffers from chronic pain at the sights of her left pleural deposits and left acetabular metastasis. She is currently being considered for external beam radiotherapy to her left hip.

## 3. Discussion

Carcinoid tumours are uncommon neuroendocrine tumours primarily affecting the gastrointestinal tract and the bronchopulmonary tree [6, 7]. They have unpredictable malignant potential with approximately one quarter of bronchial carcinoids undergoing invasive growth or metastatic spread. Overall, their slow rate of growth ensures a relatively good prognosis for bronchial carcinoids with a five-decade multicentre study reporting five-year survival rates of 73.5% [4]. 

Splenic metastasis of solid tumours is rare and is most often seen with extensive multivisceral metastatic disease from breast, lung, ovarian, gastric, and skin melanoma primaries [8]. This case presented as local disease recurrence with a solitary splenic mass on imaging although more sensitive radioactive labelled imaging later identified probable metastasis to the left acetabulum. Solitary splenic metastasis in the absence of multivisceral disease is extremely rare with Compéret et al. showing only 93 reported cases up to 2007 [9]. Of these only 10 were from lung primaries, with just 1 previous reported case of pulmonary carcinoid metastasis involving the spleen [4]. As in the current case, this previous case involved a metachronous metastasis presenting 8 years after the diagnosis of the lung primary. It is also interesting to note that in the current case, the metastases arose from a left-sided primary carcinoid. Splenic metastases have indeed been found to arise more commonly from the left lung than the right [10]. 

In view of the rarity of splenic metastasis, splenic lesions in the context of known malignant disease present a diagnostic challenge. It must be assumed that any lesion in this setting is a metastasis, but it is preferable to obtain a tissue diagnosis for confirmation as in the case presented. Despite some concerns of haemorrhagic risk from such highly vascularised tissue, ultrasound guided biopsy has been showed to be safe with comparable complication rates to biopsies of other abdominal organs [11]. 

Splenectomy for metastatic splenic lesions is generally indicated to prevent further metastatic spread, to improve the efficacy of adjuvant therapy and, therefore, improve survival, or to prevent complications such as splenic vein thrombosis, painful splenomegaly, and splenic rupture. In a review of 13 cases of splenic metastasis from lung carcinoma, Ando et al. report that out of 6 patients who did not undergo splenectomy, 4 (67%) went on to have splenic rupture with associated poor outcomes [12].

Laparoscopic splenectomy (LS) has become the standard treatment for benign and malignant haematological disorders requiring splenectomy [5]. However, there is a lack of evidence for the use of LS in the management of isolated malignant tumours of the spleen, most likely due to the rarity of the condition. The generic benefits of a laparoscopic approach to abdominal pathology apply to splenic surgery with a reduction in wound morbidity, shorter hospital stay and recovery times, and improved quality of life. The advantage of a laparoscopic approach is particularly beneficial in cases of malignancy as the associated shorter recovery times allow an earlier introduction of chemotherapeutic agents. 

Makrin et al. report their experience with LS in the treatment of 28 isolated splenic tumours and advocate its use in most cases of splenectomy for solid tumours, except in the case of substantial splenomegaly [13]. All 28 cases were treated successfully with no postoperative mortality. However, 4 cases required conversion to an open procedure to allow removal of an enlarged spleen. In cases where an enlarged spleen is encountered, a hand-assisted laparoscopic approach can also be employed to allow easier mobilisation of the spleen and resection of adjacent organs or tissues where needed [14].

## 4. Conclusion

This paper provides a further account of the rare event of metastatic carcinoid involving the spleen and the first application of a successful laparoscopic resection for its treatment. This case offers further evidence that laparoscopic splenectomy is a safe alternative to open resection in the treatment of solid organ tumours involving the spleen.
